# Survival benefits and challenges of adjuvant chemotherapy for high-grade osteosarcoma: a population-based study

**DOI:** 10.1186/s13018-023-03922-2

**Published:** 2023-06-27

**Authors:** Jinkui Wang, Mujie Li, Peng Guo, Dawei He

**Affiliations:** 1 Department of Urology, Children’s Hospital of Chongqing Medical University, 2 ZhongShan Rd, Chongqing, 400013 China; 2 Chongqing Key Laboratory of Children Urogenital Development and Tissue Engineering, Chongqing Key Laboratory of Pediatrics, Ministry of Education Key Laboratory of Child Development and Disorders, National Clinical Research Center for Child Health and Disorders, China International Science and Technology Cooperation Base of Child Development and Critical Disorders, Children’s Hospital of Chongqing Medical University, Chongqing, China; 3 Institute of Basic Medicine and Cancer (IBMC), Chinese Academy of Sciences, Hangzhou, Zhejiang China

**Keywords:** Osteosarcoma, Chemotherapy, Survival, Challenge, SEER

## Abstract

**Background:**

Osteosarcoma is the most prevalent primary malignant bone tumor. The primary treatment for osteosarcoma is a combination of chemotherapy and surgery. However, there has been no recent progress in the role of chemotherapy in improving the long-term survival of osteosarcoma patients. This study aims to analyze the factors that affect chemotherapy in patients with osteosarcoma and explore the challenges and survival benefits of chemotherapy.

**Methods:**

Patient data were downloaded from The Surveillance, Epidemiology, and End Results database. Univariable and multivariable logistic regressions were used to analyze the factors affecting patients receiving chemotherapy. Kaplan–Meier (K–M) curve was used to analyze the survival benefit of chemotherapy in patients with osteosarcoma. Finally, we used annual percentage change (APC) to evaluate the annual changes in chemotherapy treatment rates and trends in 5-year survival rates in osteosarcoma patients.

**Results:**

A total of 2157 osteosarcoma patients were included, of which 1887 patients received chemotherapy. Factors affecting patients receiving chemotherapy included age, primary tumor site, tumor size, N stage, M stage, and surgery. The K–M curve showed that older patients could benefit significantly from chemotherapy. The APC results showed no significant change in the chemotherapy treatment rate and 5-year overall survival rate of osteosarcoma patients.

**Conclusion:**

Chemotherapy is an irreplaceable treatment for patients with osteosarcoma. However, in recent years, there has been no significant progress in chemotherapy for osteosarcoma, and the long-term survival of patients has not improved significantly. New chemotherapeutic drugs or drug delivery systems are urgently needed to improve the prognosis of patients with osteosarcoma.

## Introduction

Osteosarcoma is the most common primary malignant bone tumor, which tends to occur in children and adolescents. The predilection site of osteosarcoma is long bone epiphysis, which is prone to early lung metastasis [1]. The prognosis for patients with osteosarcoma is poor, with a dismal 5-year survival rate of less than 20% before the 1970s. It was not until the emergence of chemotherapy drugs, such as methotrexate, doxorubicin and leucovorin, that the survival rate of osteosarcoma increased to more than 50% [2]. The original surgical method of osteosarcoma is mainly amputation. With the application of chemotherapy, limb-salvage surgery has become the mainstream treatment method for osteosarcoma and further improves the 5-year survival rate of patients [3]. Currently, the survival rate of patients without metastatic osteosarcoma can reach 60–70% with surgical and chemotherapy therapy, but the overall survival time of metastatic osteosarcoma is less than 30% [4]. In the past few decades, although advanced treatments have been used to improve the survival of osteosarcoma patients, the long-term survival rate has not been further improved [5].

Chemotherapy is an important treatment to improve the survival of patients with osteosarcoma. Cisplatin, doxorubicin, methotrexate and ifosfamide are first-line drugs for osteosarcoma. However, chemotherapy is unsuitable for all patients with osteosarcoma, and many factors can affect patients receiving chemotherapy. With the increase in age, the chemotherapy acceptance rate also gradually decreased. Longhi et al. [6] reviewed 43 osteosarcoma patients over 65 and found that stage, tumor volume, and surgery were important prognostic factors, but chemotherapy was not significant. Okada et al. [7] analyzed the prognosis of 64 patients with osteosarcoma over 50 years old and found that chemotherapy could not improve the prognosis of these patients. For low-grade tumors, the absence of chemotherapy can also result in high survival rates. Righi et al. [8] found that high-grade components appeared in low-grade central osteosarcoma, chemotherapy was unnecessary as long as it was less than 50%, and radical surgical resection could achieve a high survival rate. In addition, other factors may affect patients’ acceptance of chemotherapy, such as gender, race and insurance [9].

Chemotherapy regimens for osteosarcoma have been improved in recent years. A combination chemotherapy regimen of cisplatin, doxorubicin, methotrexate, and ifosfamide is used in most bone tumor treatment centers but varies from center to center. The European Osteosarcoma Intergroup (EOI) recommended the combination of cisplatin and doxorubicin (6 treatments for 18 weeks), and Memorial Sloan Kettering Cancer Center, on the other hand, uses a modified T10 regimen (eight drugs combined for 44 weeks) [10]. In addition, neoadjuvant chemotherapy also plays an important role in limb-salvage surgery. However, neoadjuvant chemotherapy did not significantly improve prognosis, and a randomized controlled trial by the Pediatric Oncology Group (POG) showed no significant difference in survival between patients receiving neoadjuvant chemotherapy and those undergoing immediate surgery after diagnosis [11]. In addition, clinical second-line chemotherapeutics such as docetaxel, gemcitabine and cyclophosphamide are also used for refractory, multi-drug-resistant and recurrent osteosarcoma. In addition, the efficacy of chemotherapy is related to a variety of factors, such as the cycle of adjuvant chemotherapy or neoadjuvant chemotherapy, the type of drugs used and the dose of drugs.

However, whether chemotherapy treatment rates for osteosarcoma have changed in recent years or 5-year survival rates have increased is unclear. In this study, we used population-based osteosarcoma patient data from The Surveillance, Epidemiology, and End Results (SEER) database to analyze the influencing factors for osteosarcoma patients receiving chemotherapy and to identify the benefit population of chemotherapy. We also analyzed changes in chemotherapy treatment rates in recent years in patients with osteosarcoma and trends in 5-year overall survival in all patients with osteosarcoma and patients receiving chemotherapy. This study aimed to determine the survival benefits of adjuvant chemotherapy in different populations of osteosarcoma and to explore current challenges to chemotherapy.

## Method

### Data source and study population

The SEER*Stat software (version 8.3.9) was used to obtain information on the clinical characteristics and treatment status of patients diagnosed with osteosarcoma in a cohort from 2000 to 2019. The SEER database is an open-access public database that collects tumor patient data from 18 cancer registries covering approximately 30% of the US population. All patient identifying information in this database is anonymous, so we do not require informed consent from patients for this study.

**Fig. 1:**
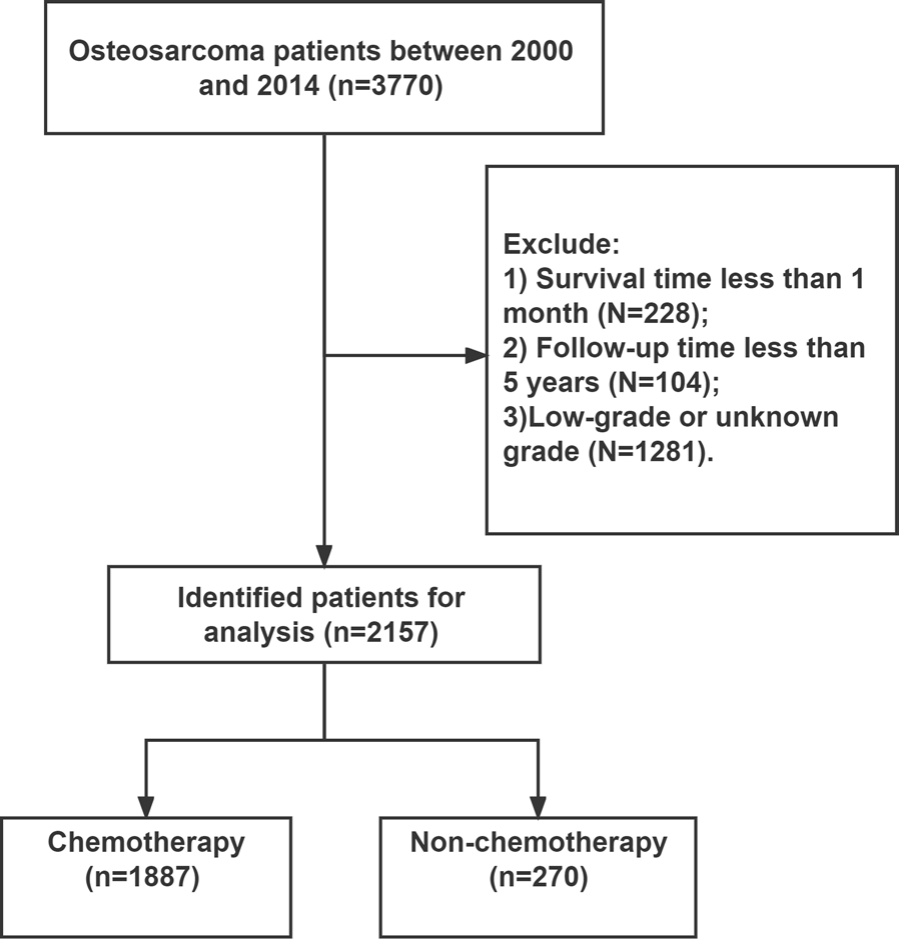
Flowchart for inclusion and exclusion of osteosarcoma patients

To obtain complete 5-year survival information, we included only patients from 2000 to 2014. Inclusion criteria: (1) patients diagnosed with osteosarcoma; (2) the year of diagnosis was 2000–2014. Exclusion criteria: (1) patients who were followed up for less than 5 years; (2) patients whose survival time is less than 1 month (patients who may die from surgery or other causes); (3) Low-grade or grade unknown osteosarcoma. The flowchart of inclusion and exclusion of osteosarcoma patients is shown in Fig. 1.

### Univariable and multivariable logistic regression analysis

To analyze the factors influencing patients’ choice of chemotherapy, univariable and multivariable logistic regression analyses were used. Variables included age, sex, marital status, mean household income, urban or rural residence, race, primary tumor site, tumor laterality, *T* stage, *N* stage, *M* stage, surgery, radiotherapy, tumor size, and tumor number.

### Kaplan–Meier curve analysis

We first used the Kaplan–Meier (K–M) curve to determine the survival benefit of chemotherapy in patients with osteosarcoma. We used the K–M curve to analyze the survival benefits of chemotherapy in different groups, including age groups (0–60 and > 60 years old), sex (male and female), and high grade (III/IV)), and M stage (M0/M1).

### Univariable and multivariable Cox regression analysis

We analyzed the prognostic factors affecting high-grade osteosarcoma patients. Initially, we included all variables in a univariable Cox regression analysis, including age, sex, marital status, mean household income, urban or rural residence, race, primary tumor site, tumor laterality, T stage, N stage, M stage, surgery, radiotherapy, tumor size, and tumor number. Subsequently, the factors with a *p* value less than 0.05 were included in a multivariable Cox regression analysis. The final multivariable Cox regression analysis revealed the independent risk factors for patient prognosis.

### Trends in chemotherapy treatment rate and survival rate over time

We calculated the annual rate of chemotherapy treatment for osteosarcoma patients from 2000 to 2014. We also calculated the 5-year survival rate for patients with new osteosarcoma per year based on their year of diagnosis and the 5-year survival rate for patients receiving chemotherapy. To describe the change in chemotherapy treatment rates and 5-year survival rates over time, we calculated the annual percentage change (APC), representing the average annual increase/decrease in treatment rates and survival rates.

### Statistical analysis

All variables were described by frequency, and comparison between groups was performed by chi-square test. Univariable and multivariable logistic regression models were used to analyze the influencing factors of chemotherapy use. K–M curves and log-rank tests were used to compare survival differences between groups. APC was used to analyze annual trends in chemotherapy treatment and survival rates. SPSS26.0 and R4.1.0 were used for all statistical analyses, and *p* values less than 0.05 were considered statistically significant.

## Result

### Clinical features

A total of 2157 patients with osteosarcoma were enrolled in this study, of whom 1887 received chemotherapy. The demographic information, clinicopathological information and follow-up results of all patients are shown in Table 1. It can be seen from the table that the rate of chemotherapy treatment is lower in older patients (*p* < 0.001); single patients had a higher rate of chemotherapy treatment (*p* < 0.001), which may be related to the fact that the majority of single patients were children and adolescents. Patients with osteosarcoma whose primary sites were bones of the skull, face, mandible, and associated joints received lower rates of chemotherapy than those with other sites (*p* < 0.001); patients with lower TNM stage also had lower chemotherapy treatment rates (*p* < 0.001); chemotherapy treatment rates were significantly lower in patients who did not receive surgery (*p* < 0.001); patients receiving radiotherapy were less likely to receive concurrent chemotherapy (*p* < 0.001); patients with multiple tumors received more chemotherapy (*p* < 0.001).

**Table 1:** Clinicopathological characteristics of patients with osteosarcoma

	ALL *N* = 2157	Chemotherapy *N* = 1887	Non-chemotherapy *N* = 270	*p*
Age				< 0.001
0–10	253 (11.7%)	246 (13.0%)	7 (2.59%)	
11–20	955 (44.3%)	933 (49.4%)	22 (8.15%)	
21–40	394 (18.3%)	349 (18.5%)	45 (16.7%)	
41–60	323 (15.0%)	244 (12.9%)	79 (29.3%)	
> 60	232 (10.8%)	115 (6.09%)	117 (43.3%)	
Sex				0.772
Male	1212 (56.2%)	1063 (56.3%)	149 (55.2%)	
Female	945 (43.8%)	824 (43.7%)	121 (44.8%)	
Race				0.816
White	1617 (75.0%)	1413 (74.9%)	204 (75.6%)	
Black	321 (14.9%)	284 (15.1%)	37 (13.7%)	
Other	219 (10.2%)	190 (10.1%)	29 (10.7%)	
Marital				< 0.001
Married	503 (23.3%)	378 (20.0%)	125 (46.3%)	
Single	1501 (69.6%)	1423 (75.4%)	78 (28.9%)	
Divorced, separated, widowed, unmarried or domestic partner or unknown	153 (7.09%)	86 (4.56%)	67 (24.8%)	
Year of diagnosis1				0.619
2000–2002	571 (26.5%)	490 (26.0%)	81 (30.0%)	
2003–2005	405 (18.8%)	358 (19.0%)	47 (17.4%)	
2006–2008	447 (20.7%)	391 (20.7%)	56 (20.7%)	
2009–2011	435 (20.2%)	387 (20.5%)	48 (17.8%)	
2012–2014	299 (13.9%)	261 (13.8%)	38 (14.1%)	
Median household income				0.635
> $74,999	556 (25.8%)	480 (25.4%)	76 (28.1%)	
$60,000– $74,999	982 (45.5%)	863 (45.7%)	119 (44.1%)	
< $60,000	619 (28.7%)	544 (28.8%)	75 (27.8%)	
Rural and urban				0.401
Counties in metropolitan areas ge 1 million pop	1356 (62.9%)	1193 (63.2%)	163 (60.4%)	
Other	801 (37.1%)	694 (36.8%)	107 (39.6%)	
Primary site				< 0.001
Limbs	1679 (77.8%)	1549 (82.1%)	130 (48.1%)	
Pelvic bones, sacrum, coccyx and associated joints	176 (8.16%)	133 (7.05%)	43 (15.9%)	
Bones of skull and face and associated joints/Mandible	191 (8.85%)	131 (6.94%)	60 (22.2%)	
Vertebral column/Rib, sternum, clavicle and associated joints	98 (4.54%)	67 (3.55%)	31 (11.5%)	
Unknown	13 (0.60%)	7 (0.37%)	6 (2.22%)	
Laterality				< 0.001
Left	940 (43.6%)	849 (45.0%)	91 (33.7%)	
Right	958 (44.4%)	864 (45.8%)	94 (34.8%)	
Not a paired site or unknown	259 (12.0%)	174 (9.22%)	85 (31.5%)	
T				< 0.001
T1	556 (25.8%)	485 (25.7%)	71 (26.3%)	
T2	770 (35.7%)	705 (37.4%)	65 (24.1%)	
T3	53 (2.46%)	46 (2.44%)	7 (2.59%)	
TX	778 (36.1%)	651 (34.5%)	127 (47.0%)	
N				0.001
N0	1459 (67.6%)	1304 (69.1%)	155 (57.4%)	
N1	39 (1.81%)	34 (1.80%)	5 (1.85%)	
NX	659 (30.6%)	549 (29.1%)	110 (40.7%)	
M				0.045
M0	1237 (57.3%)	1096 (58.1%)	141 (52.2%)	
M1	322 (14.9%)	285 (15.1%)	37 (13.7%)	
MX	598 (27.7%)	506 (26.8%)	92 (34.1%)	
Surgery				< 0.001
No	284 (13.2%)	209 (11.1%)	75 (27.8%)	
Yes	1873 (86.8%)	1678 (88.9%)	195 (72.2%)	
Radiation				< 0.001
No	1940 (89.9%)	1734 (91.9%)	206 (76.3%)	
Yes	217 (10.1%)	153 (8.11%)	64 (23.7%)	
Tumor size				< 0.001
< 60 mm	330 (15.3%)	275 (14.6%)	55 (20.4%)	
60–120 mm	683 (31.7%)	618 (32.8%)	65 (24.1%)	
> 120 mm	362 (16.8%)	335 (17.8%)	27 (10.0%)	
Unknown	782 (36.3%)	659 (34.9%)	123 (45.6%)	
Total number of in tumors				< 0.001
Single	1825 (84.6%)	1647 (87.3%)	178 (65.9%)	
Multiple	332 (15.4%)	240 (12.7%)	92 (34.1%)	
Survival months	85.3 (70.9)	90.1 (70.9)	52.4 (61.5)	< 0.001
Status				< 0.001
Dead	1123 (52.1%)	915 (48.5%)	208 (77.0%)	
Alive	1034 (47.9%)	972 (51.5%)	62 (23.0%)	

### Univariable and multivariable logistic regression analysis

We first incorporated all variables into a univariable logistic regression analysis to determine the factors affecting patients receiving chemotherapy. The results showed age, marital status, primary tumor site, tumor laterality, T stage, N stage, M stage, surgery, radiotherapy, tumor size, and tumor number were influencing factors for patients receiving chemotherapy. These factors were then incorporated into the multivariable logistic regression model to determine independent influencing factors. The results showed that age, primary tumor site, tumor size, N stage, M stage, and surgery were independent factors affecting chemotherapy patients. Older patients are less likely to receive chemotherapy, single patients are more likely to receive chemotherapy, and patients with primary sites such as the skull, face and mandible are less likely to receive chemotherapy. N1 and M1 were more susceptible to chemotherapy. Patients who have surgery are more likely to receive chemotherapy. The results of the logistic regression analysis are shown in Table 2.

**Table 2:** Univariable and multivariable logistic regression models in patients with osteosarcoma predicting chemotherapy

	Univariable				Multivariable			
	HR	95% CI		*p*	HR	95% CI		*p*
Age								
0–10 year	Reference				Reference			
11–20 year	1.21	0.51	2.86	0.67	1.217	0.512	2.894	0.656
21–40 year	0.22	0.1	0.5	< 0.001	0.258	0.111	0.6	0.002
41–60 year	0.09	0.0	0.19	< 0.001	0.113	0.047	0.272	< 0.001
> 60year	0.03	0.01	0.06	< 0.001	0.039	0.016	0.097	< 0.001
Sex								
Male	Reference							
Female	0.95	0.74	1.23	0.72				
Race								
White	Reference							
Black	1.11	0.76	1.61	0.59				
Other	0.95	0.62	1.44	0.79				
Marital								
Married	Reference				Reference			
Single	0.17	0.12	0.22	< 0.001	1.11	0.727	1.695	0.628
Divorced, Separated, Widowed, Unmarried or Domestic Partner or unknown	0.07	0.05	0.1	< 0.001	0.656	0.389	1.106	0.114
Mean family income								
> $74,999	Reference							
$60,000–$74,999	1.15	0.84	1.56	0.38				
< $60,000	1.15	0.82	1.62	0.43				
Rural or urban								
Counties in metropolitan areas ge 1 million pop	Reference							
Other	1.13	0.87	1.47	0.36				
Primary site								
Limbs	Reference				Reference			
Pelvic bones, sacrum, coccyx and associated joints	0.26	0.18	0.38	< 0.001	0.855	0.534	1.37	0.516
Bones of skull and face and associated joints/Mandible	0.18	0.13	0.26	< 0.001	0.445	0.296	0.669	< 0.001
Vertebral column/Rib, sternum, clavicle and associated joints	0.18	0.11	0.29	< 0.001	0.479	0.281	0.816	0.007
Unknown	0.1	0.03	0.3	< 0.001	0.379	0.101	1.422	0.151
Laterality								
Left	Reference							
Right	0.99	0.73	1.33	0.92				
Not a paired site or unknown	0.22	0.16	0.31	< 0.001				
T								
T1								
T2	1.59	1.11	2.27	0.01				
T3	0.96	0.42	2.21	0.93				
TX	0.75	0.55	1.03	0.07				
N								
N0	Reference				Reference			
N1	0.81	0.31	2.1	0.66	1.035	0.352	3.037	0.951
NX	0.59	0.46	0.77	< 0.001	0.291	0.146	0.58	< 0.001
M								
M0	Reference				Reference			
M1	0.99	0.67	1.46	0.96	1.446	0.88	2.376	0.146
MX	0.71	0.53	0.94	0.02	2.071	1.005	4.268	0.048
Surgery								
No	Reference				Reference			
Yes	3.09	2.28	4.18	< 0.001	1.614	1.082	2.41	0.019
Radiation								
No	Reference							
Yes	0.28	0.21	0.39	< 0.001				
Tumor size								
< 60 mm	Reference							
60–120 mm	1.9	1.29	2.8	< 0.001				
> 120 mm	2.48	1.52	4.04	< 0.001				
Unknown	1.07	0.76	1.52	0.7				
Total number of tumors								
Single	Reference							
Multiple	0.28	0.21	0.38	< 0.001				

### Kaplan–Meier curve analysis

**Fig. 2:**
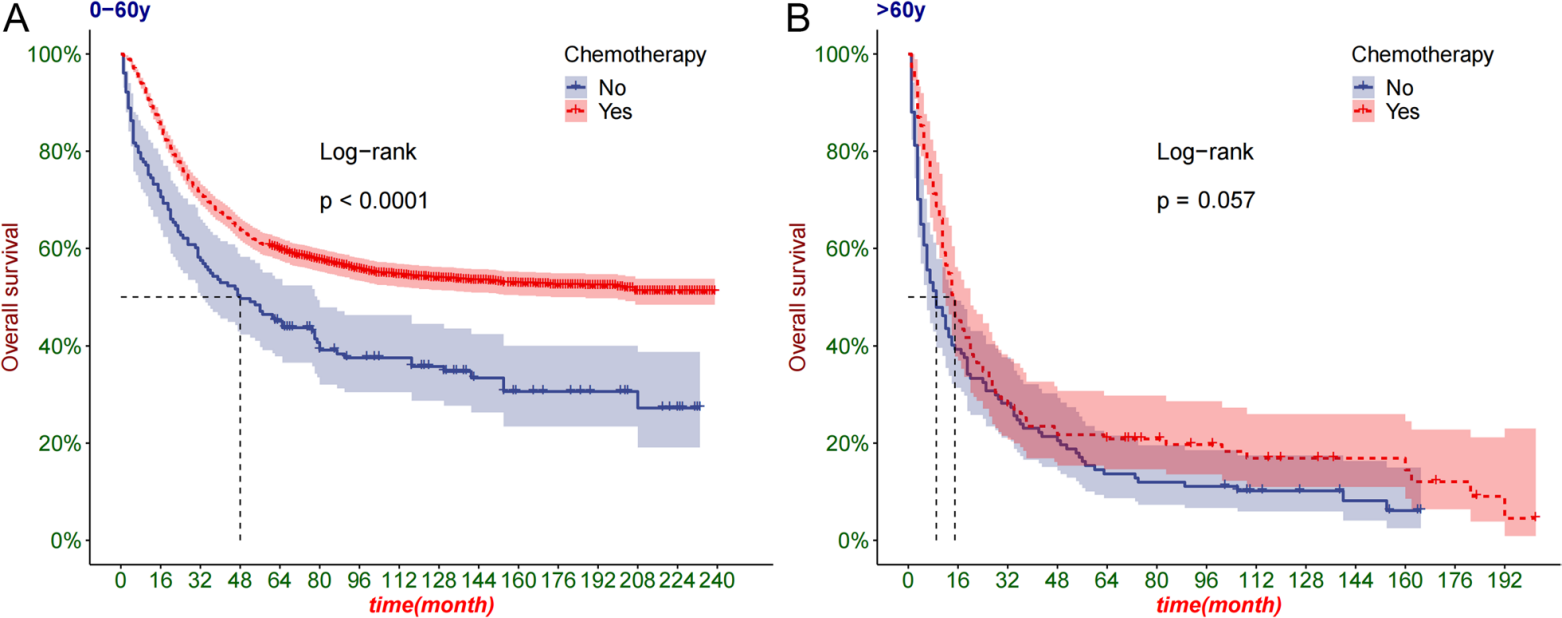
The K-M curve of patients grouped by age. **A** The K–M curve of patients aged 0–60; **B** The K–M curve of patients aged over 60

**Fig. 3:**
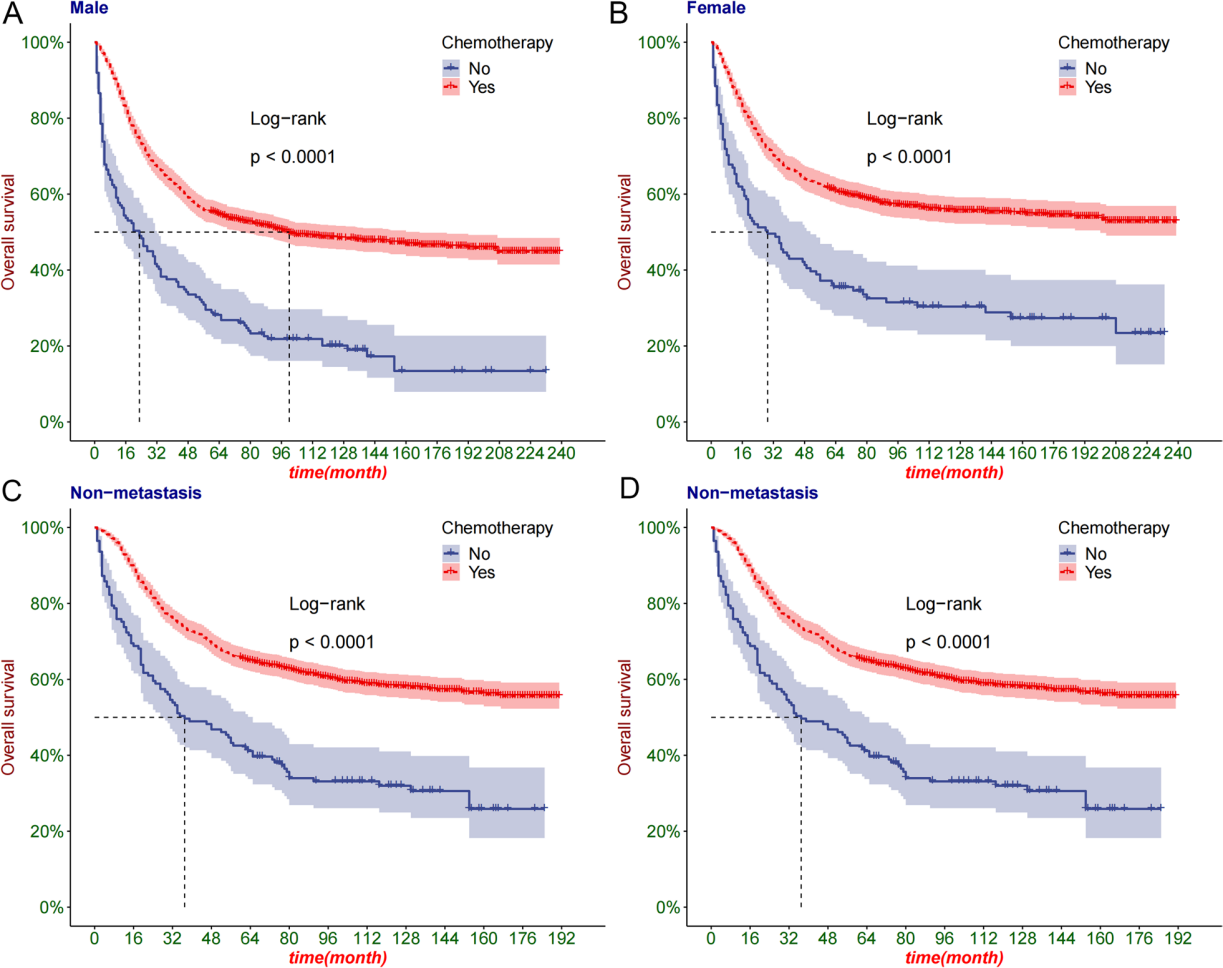
The K-M curve of patients grouped by sex and M stage. **A** The K–M curve of male patients; **B** The K–M curve of female patients; **C** K–M curve of M0 tumor patients; **D** K–M curve of M1 tumor patients

We analyzed the benefit of chemotherapy in different groups of patients. The age group showed that the survival rate of patients aged 0–60 years was significantly lower in the chemotherapy group, but there was no significant difference between the two groups of patients over 60 years old (Fig. 2A–B). Sex grouping showed that chemotherapy improved prognosis in men and women (Fig. 3A, B). In patients with osteosarcoma with distant metastases and non-metastatic, chemotherapy significantly improved survival (Fig. 3C, D).

### Univariable and multivariable Cox regression analysis

In order to analyze the factors influencing patient prognosis, we conducted both univariable and multivariable Cox regression analyses. The univariable Cox regression analysis revealed that age, sex, marital status, primary tumor site, TNM staging, surgery, radiotherapy, chemotherapy, tumor size, and tumor number were factors affecting patient survival. The multivariable Cox regression analysis showed that age, sex, primary tumor site, TNM staging, surgery, radiotherapy, chemotherapy, and tumor size were independent risk factors influencing patient survival (Table 3). The results indicated that chemotherapy was a significant protective factor, confirming that in high-grade osteosarcoma patients, chemotherapy can significantly improve patient survival.

**Table 3:** Univariable and multivariable Cox regression models in patients with osteosarcoma predicting overall survival

	Univariable				Multivariable			
	HR	95% CI		*p*	HR	95% CI		*p*
Age								
0–10 year	Reference				Reference			
11–20 year	1.29	1.02	1.62	0.034	1.193	0.945	1.506	0.139
21–40 year	1.6	1.24	2.06	< 0.001	1.534	1.183	1.989	0.001
41–60 year	3.11	2.43	3.98	< 0.001	2.517	1.941	3.263	< 0.001
> 60 year	5.92	4.6	7.62	< 0.001	3.897	2.951	5.146	< 0.001
Sex								
Male	Reference				Reference			
Female	0.82	0.73	0.93	0.001	0.805	0.712	0.91	0.001
Race								
White	Reference							
Black	1.04	0.88	1.22	0.644				
Other	0.89	0.72	1.09	0.246				
Marital								
Married	Reference							
Single	2.08	1.83	2.37	< 0.001				
Divorced, Separated, Widowed, Unmarried or Domestic Partner or unknown	2.67	2.19	3.26	< 0.001				
Mean family income								
> $74,999	Reference							
$60,000–$74,999	0.92	0.79	1.06	0.232				
< $60,000	0.87	0.74	1.01	0.074				
Rural or urban								
Counties in metropolitan areas ge 1 million pop	Reference							
Other	0.98	0.87	1.11	0.751				
Primary site								
Limbs	Reference				Reference			
Pelvic bones, sacrum, coccyx and associated joints	3.37	2.82	4.02	< 0.001	1.948	1.6	2.373	< 0.001
Bones of skull and face and associated joints/Mandible	1.7	1.4	2.05	< 0.001	1.509	1.21	1.881	< 0.001
Vertebral column/Rib, sternum, clavicle and associated joints	3.01	2.38	3.8	< 0.001	2.202	1.704	2.845	< 0.001
Unknown	4.96	2.8	8.78	< 0.001	2.751	1.53	4.947	0.001
Laterality								
Left	Reference							
Right	0.98	0.86	1.11	0.712				
Not a paired site or unknown	2.03	1.71	2.4	< 0.001				
T								
T1					Reference			
T2	1.23	1.05	1.44	0.012	0.959	0.764	1.205	0.72
T3	2.66	1.92	3.7	< 0.001	1.696	1.185	2.429	0.004
TX	1.4	1.2	3.7	< 0.001	0.801	0.468	1.37	0.418
N								
N0	Reference				Reference			
N1	3.74	2.66	5.25	< 0.001	1.76	1.225	2.53	0.002
NX	1.14	1	1.29	0.044	0.993	0.734	1.344	0.963
M								
M0	Reference				Reference			
M1	2.98	2.56	3.47	< 0.001	2.719	2.288	3.231	< 0.001
MX	1.29	1.12	1.48	< 0.001	1.051	0.739	1.494	0.783
Surgery								
No	Reference				Reference			
Yes	0.29	0.25	0.34	< 0.001	0.498	0.423	0.587	< 0.001
Radiation								
No	Reference				Reference			
Yes	2.68	2.28	3.15	< 0.001	1.235	1.026	1.486	0.026
Chemotherapy								
No	Reference				Reference			
Yes	0.42	0.36	0.49	< 0.001	0.815	0.685	0.969	0.02
Tumor size								
< 60 mm	Reference				Reference			
60–120 mm	1.23	1.01	1.5	0.042	1.572	1.231	2.008	< 0.001
> 120 mm	1.57	1.26	1.94	< 0.001	1.982	1.456	2.698	< 0.001
Unknown	1.49	1.23	1.81	< 0.001	2.315	1.348	3.975	0.002
Total number of tumors								
Single	Reference							
Multiple	1.9	1.65	2.19	< 0.001				

### Trends in chemotherapy rate and survival rate over time

**Fig. 4:**
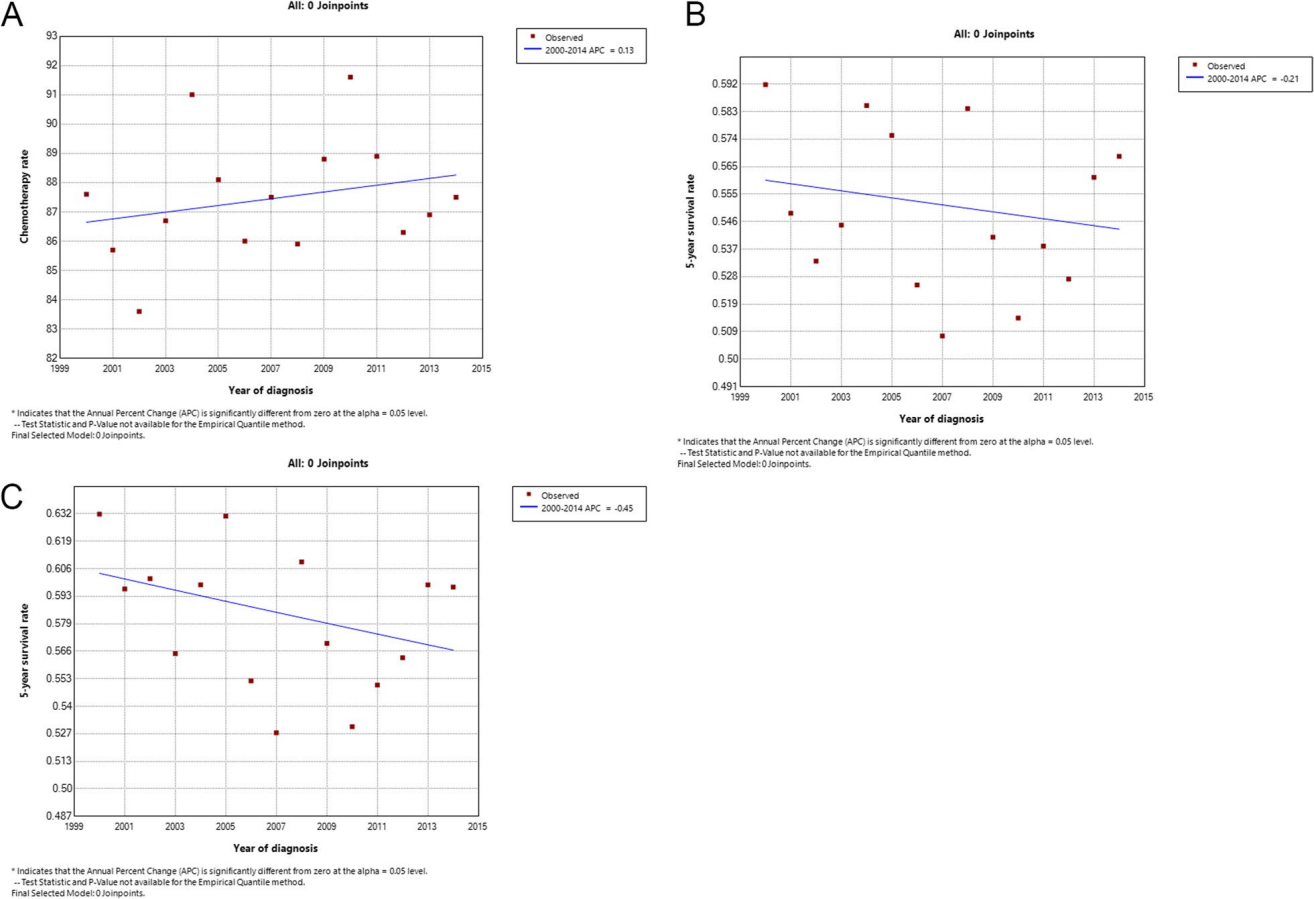
**A** The annual chemotherapy treatment rate of osteosarcoma; **B** trends in annual 5-year overall survival of osteosarcoma; **C** the trend of annual 5-year overall survival rate of osteosarcoma receiving chemotherapy

We analyzed annual trends in chemotherapy treatment rates for all osteosarcoma patients from 2000 to 2014. The results showed that the rate of chemotherapy treatment increased over the 15 years (APC, 0.13%) but was not significant (Fig. 4A). We then analyzed 5-year overall survival rate for patients with osteosarcoma over the last 15 years and 5-year overall survival rate for patients receiving chemotherapy. Results showed an upward trend in 5-year overall survival (APC, − 0.21%), but insignificant (Fig. 4B). The 5-year overall survival of patients receiving chemotherapy showed a downward trend (APC, − 0.45%) and was insignificant (Fig. 4C).

## Discussion

Although osteosarcoma is the most common bone tumor, its incidence is still low, about 3 per 1 million [12]. There has been little progress in treating osteosarcoma since adjuvant chemotherapy began in the 1970s [13]. Due to off-target effects and uncontrolled release, powerful anticancer drugs have limited dosage and low patient compliance. Many studies are currently aimed at developing new chemotherapy agents for osteosarcoma to improve patient survival, but the results have been unsatisfactory. A Phase 3 trial using zoledronate sodium in combination with chemotherapy and surgery for osteosarcoma did not yield favorable results [14]. Although many studies have used drug delivery systems to control the release of chemotherapy drugs to reduce their side effects, most of them are still confined to preclinical studies [15]. Therefore, new treatment strategies and new chemotherapy drugs are needed, and clinical trials must be conducted to find more promising treatments.

This study analyzed the differences between patients who received chemotherapy for osteosarcoma and those who did not. In both difference analysis and influence factor analysis, we found that age was an important factor. In younger patients, osteosarcoma is more likely to occur in the distal femur, proximal tibia, and humerus, whereas, in older patients, osteosarcoma often occurs in the axial bone [16]. The efficacy of adjuvant chemotherapy in middle-aged and elderly patients with human osteosarcoma differs. Bacci et al. [17] found that adjuvant chemotherapy was effective for patients over 40 with high-grade limb osteosarcoma. Grimer et al. also reported 238 cases of non-metastatic high-grade osteosarcoma in the extremities aged 40–60. The survival rate in the chemotherapy group was significantly higher than that in the non-chemotherapy group [18]. In contrast, some studies have found no significant prognostic benefit of adjuvant chemotherapy in middle-aged and elderly patients with osteosarcoma. Iwata et al. reported on 86 patients over 40 with high-grade osteosarcoma and found that adjuvant chemotherapy did not significantly improve 5-year survival [19]. In our study, osteosarcoma patients under 60 years of age who received chemotherapy had a lower survival rate, which may be due to selection bias, because patients with advanced stage or higher malignancy are more likely to receive chemotherapy. In addition, there was no significant difference in survival rate between chemotherapy group and non-chemotherapy group in patients over 60 years old, indicating that chemotherapy could not improve the survival of elderly patients. Although the SEER database does not provide details of chemotherapy, such as chemotherapy regimens and dosages, poor prognosis after chemotherapy in elderly patients should be associated with adverse chemotherapy reactions. In middle-aged and elderly patients, chemotherapy often leads to myelosuppression and renal toxicity [20]. Therefore, adjuvant chemotherapy should be carefully selected for elderly patients with osteosarcoma.

In addition, tumor grade is also a factor influencing chemotherapy in patients with osteosarcoma, and survival analysis shows that patients with high-grade osteosarcoma can significantly benefit from adjuvant chemotherapy. The onset of multi-drug chemotherapy has significantly improved the prognosis of patients with high-grade osteosarcoma [21]. Previous studies have shown that adjuvant chemotherapy combined with surgery can improve the survival rate of patients with high-grade osteosarcoma from 10–20% to more than 60% compared with surgery alone [22, 23]. In our study, most patients with high-grade osteosarcoma received chemotherapy and significantly extended their survival compared to the non-chemotherapy group. Therefore, our findings suggest that adjuvant chemotherapy is an effective treatment for patients with high-grade osteosarcoma and that adequate chemotherapy should be given simultaneously with surgery.

The Cox regression analysis revealed that female patients were a protective factor for survival. These results indicate that there may be sex differences in the treatment of osteosarcoma. The treatment rate of women has also been reported in other diseases or tumors. Rose et al. reported the difference in the chemotherapy treatment rate of 23,981 patients with advanced bladder cancer, and the results showed that the chemotherapy treatment rate of women was significantly lower than that of men, and the overall survival rate of women was significantly lower than that of men [24]. Similarly, some studies have found that women are significantly less likely than men to use aspirin and statins to treat cardiovascular disease [25, 26]. However, these studies did not specify a direct relationship between treatment rates and survival. Therefore, further research is needed to explore the low treatment and survival rates in women with osteosarcoma.

The primary site of the tumor is also a factor influencing the rate of chemotherapy, especially the low rate of chemotherapy in patients with cranial and facial osteosarcoma. Because the risk of distant metastasis of craniofacial osteosarcoma has been considered low by previous studies, the role of chemotherapy is unclear [27, 28]. However, Salvati et al. found that chemotherapy could reduce low local recurrence and metastasis of cranial osteosarcoma [29]. Therefore, chemotherapy is an indispensable treatment for craniofacial osteosarcoma. In addition, our study also found that the later the tumor stage, the larger the tumor size, and the more important the role of chemotherapy. Adjuvant chemotherapy can significantly improve the prognosis of patients with advanced osteosarcoma.

However, there are many factors that can influence the effectiveness of chemotherapy in patients, such as the chemotherapy cycle, dosage, and drugs used. Min et al. [30] collected data from 333 patients with high-grade osteosarcoma and found that the chemotherapy cycle significantly affects patient survival. The median survival time for patients with a chemotherapy cycle of less than 4 cycles was 20 months, while it was 57 months for patients with a chemotherapy cycle of more than 4 cycles. Additionally, patients who responded well to chemotherapy had significantly higher survival rates. Huvos et al. [31] quantified the degree of tumor necrosis in patient tumor specimens, where a tumor necrosis rate of > 90% was defined as high necrosis, and < 90% was defined as low necrosis. The degree of tumor necrosis is an important factor in evaluating the effectiveness of chemotherapy.

We analyzed time trends in chemotherapy treatment rates for all osteosarcoma patients over the last 15 years. Although the results showed a slight upward trend in the rate of chemotherapy for osteosarcoma, it was still not significant. At the same time, we analyzed the time change trend of 5-year survival for all patients and patients receiving chemotherapy over the 15 years. There was no significant change in survival for patients. These results indicate that adjuvant chemotherapy for osteosarcoma has not progressed significantly in the past 15 years. Further research on new drugs or drug delivery systems is urgently needed to increase the effectiveness of chemotherapy treatment and thus improve the prognosis of patients with osteosarcoma.

The study still has some limitations. First, this study is a retrospective analysis, and there is still some bias in patient selection. Second, the lack of comorbidities and insurance data in the SEER database may influence the outcome of chemotherapy choices and survival differences. The SEER database lacks specific parameters regarding patient chemotherapy, such as chemotherapy cycles, drugs, and dosages, which are crucial factors in assessing prognosis after chemotherapy. Additionally, we also lack variables to assess the degree of tumor necrosis, which is important for evaluating chemotherapy effectiveness. Therefore, we need to conduct single-center studies to collect chemotherapy parameters from patients in order to assess the role of chemotherapy in osteosarcoma. Finally, it should be noted that our conclusions are based on retrospective research, and further confirmation is required through prospective studies.

## Conclusion

We analyzed the factors influencing chemotherapy treatment in patients with osteosarcoma. The results showed that age, primary tumor site, tumor size, N stage, M stage, and surgery influenced chemotherapy treatment. We then analyzed the benefits of adjuvant chemotherapy in patients with osteosarcoma. The results indicate that younger patients benefit more from chemotherapy. In addition, we analyzed the changes in chemotherapy treatment rate and 5-year overall survival rate of patients over the past 15 years. The results showed no significant progress in the chemotherapy of osteosarcoma, suggesting that the treatment of osteosarcoma still faces great challenges.
