# Correction: Survival benefits and challenges of adjuvant chemotherapy for high-grade osteosarcoma: a population-based study

**DOI:** 10.1186/s13018-023-04326-y

**Published:** 2023-11-06

**Authors:** Jinkui Wang, Mujie Li, Peng Guo, Dawei He

**Affiliations:** 1 Department of Urology, Children’s Hospital of Chongqing Medical University, 2 ZhongShan Rd, Chongqing, 400013 China; 2 Chongqing Key Laboratory of Children Urogenital Development and Tissue Engineering, Chongqing Key Laboratory of Pediatrics, Ministry of Education Key Laboratory of Child Development and Disorders, National Clinical Research Center for Child Health and Disorders, China International Science and Technology Cooperation Base of Child Development and Critical Disorders, Children’s Hospital of Chongqing Medical University, Chongqing, China; 3 Institute of Basic Medicine and Cancer (IBMC), Chinese Academy of Sciences, Hangzhou, Zhejiang China

**Correction: Journal of Orthopaedic Surgery and Research (2023) 18:465** 10.1186/s13018-023-03922-2

Following publication of the original article [1], Following publication of the original article [1], the authors identified an error in Fig. 3D.

The authors apologize for the error, but the change does not affect the current findings. The Fig. 3D has been updated and the original article [1] has been corrected.

**Fig. 3:**
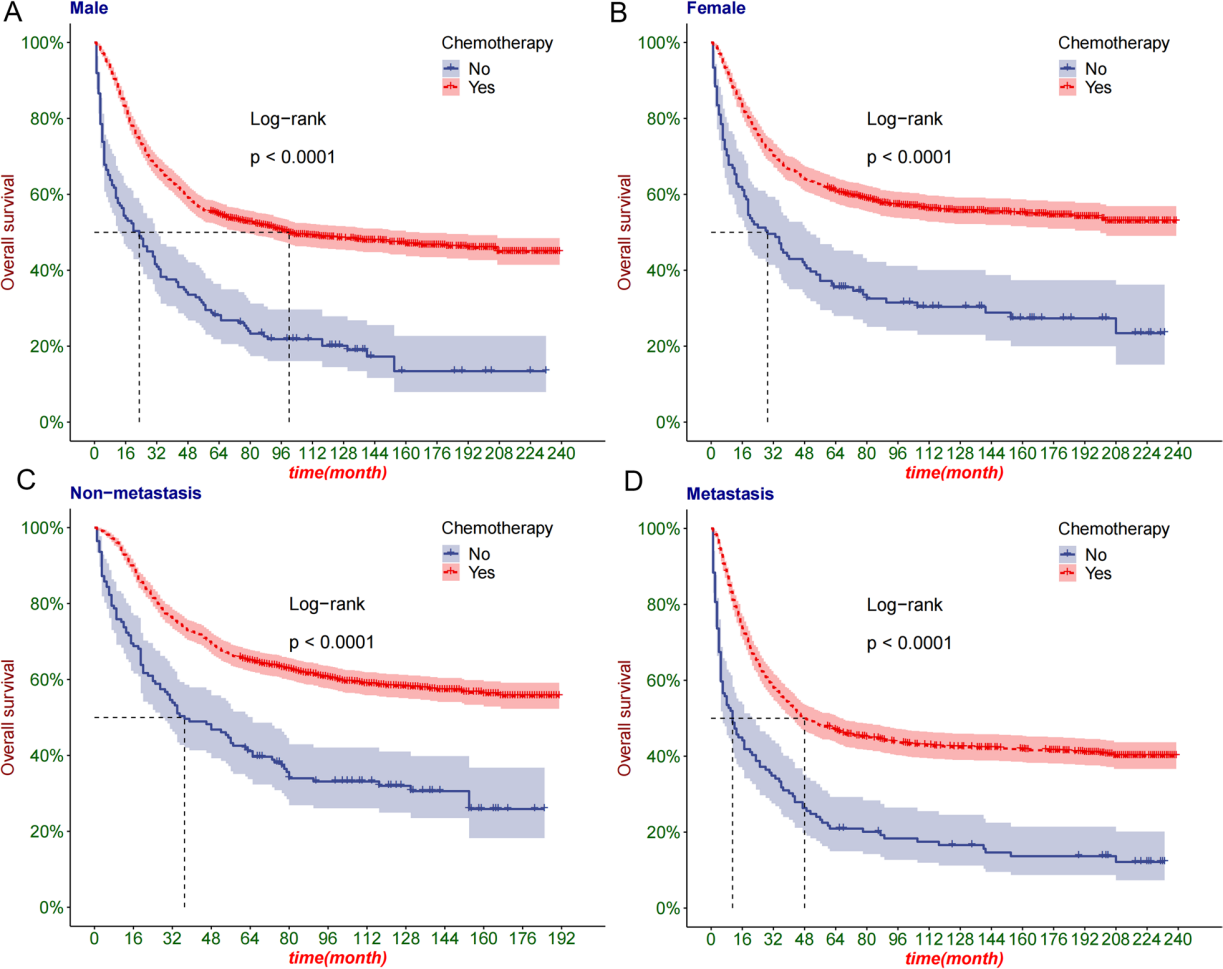
The K–M curve of patients grouped by sex and M stage. **A** The K–M curve of male patients; **B** The K–M curve of female patients; **C** K–M curve of M0 tumor patients; **D** K–M curve of M1 tumor patients
